# IL‐6/YAP1/β‐catenin signaling is involved in intervertebral disc degeneration

**DOI:** 10.1002/jcp.27065

**Published:** 2018-12-03

**Authors:** Jian Chen, Zhengfeng Mei, Bao Huang, Xuyang Zhang, Junhui Liu, Zhi Shan, Jiasheng Wang, Xianjun Wang, Fengdong Zhao

**Affiliations:** ^1^ Department of Orthopaedics Sir Run Run Shaw Hospital, School of Medicine, Zhejiang University Hangzhou China; ^2^ Department of Orthopaedics The Third People Hospital of Hangzhou Hangzhou China; ^3^ Department of Orthopaedics Linhai Second People's Hospital Taizhou China

**Keywords:** β‐catenin, intervertebral disc degeneration (IDD), nucleus pulposus (NP), yes‐associated protein 1 (YAP1)

## Abstract

Yes‐associated protein 1 (YAP1) is a transcriptional coactivator and negative regulator of the Hippo pathway. It regulates diverse cellular processes, such as cell proliferation, contact inhibition, and tissue size. However, the role of YAP1 in intervertebral disc degeneration (IDD) remains elusive. Here, we demonstrated that YAP1 was activated by Interleukin 6 (IL‐6) through tyrosine phosphorylation in nucleus pulposus cells (NP cells). Overexpression of YAP1 decreased Sox‐9, Col‐II, aggrecan expression, whereas increased matrix metalloproteinases 13 level. In contrast, knockdown of YAP1 by small interfering RNA (siRNA) showed opposite effects and rescued IL‐6 induced NP cells degeneration. In addition, western blot showed that IL‐6 treatment increased YAP1 and β‐catenin protein level; co‐immunoprecipitation (Co‐IP) and immunofluorescence analysis showed that IL‐6 enhanced YAP1 and β‐catenin interaction and nuclear accumulation. Knockdown of β‐catenin by siRNA blocked IL‐6 treatment or YAP1 overexpression induced degeneration. Moreover, we found that verteporfin, a specific inhibitor of YAP1, effectively alleviated IDD development in rat disks. Taken together, our findings indicated that YAP1 plays an important role in IDD, and β‐catenin is essential for IL‐6/YAP1 signaling.

## 1 INTRODUCTION

Intervertebral disc degeneration (IDD) is a major cause of low back pain and various degenerative spinal disorders. IDD has been a global health issue, which places a heavy burden on the healthcare system and results in severe economic consequences (Martin et al., 2008). The main pathological changes that occur in IDD include proteolytic degradation of the extracellular matrix (ECM). Interleukin 6 (IL‐6) plays an important role as a proinflammatory cytokine in the development of IDD by promoting ECM degradation. It has been reported that the serum level of IL‐6 is upregulated in patients with IDD and is associated with low back pain (Deng, Zhao, Kang, & Zhang, 2016; Weber et al., 2016). IL‐6 activates signal transducers and activators of transcription 3 (STAT3) in nucleus pulposus cells (NP cells) and annulus fibrosus cells to induce IDD by increasing the matrix metalloproteinases (MMPs) expression (Ji et al., 2016; Suzuki et al., 2016). However, the molecular mechanism of IL‐6‐induced IDD is not fully understood.

Yes‐associated protein 1 (YAP1) is a transcriptional coactivator and negative regulator of the Hippo pathway. It regulates diverse cellular processes, such as cell proliferation, contact inhibition, and tissue size (Zhao et al., 2007). Recent studies showed that YAP1 is a negative regulator of chondrocyte differentiation of mesenchymal stem cells; downregulation of YAP1 was required for chondrogenesis (Karystinou et al., 2015). YAP1 regulates chondrocyte differentiation at multiple steps, in which it promotes early chondrocyte proliferation but inhibits the expression of Col‐II and aggrecan (Deng et al., 2016). For NP cells sharing similar phenotype with chondrocyte, we hypothesized that YAP1 may play an important role in NP cells.

β‐Catenin signaling plays a critical role in IDD progression. β‐Catenin protein is upregulated in disc tissue samples from patients with IDD; β‐catenin conditional activation in mice showed severe defects in intervertebral disks and the expression of MMP‐13 and a disintegrin and metalloproteinase with thrombospondin motifs (ADAMTS‐5) were increased (Wang et al., 2012). Proinflammatory cytokines IL‐1β and tumor necrosis factor‐α (TNF‐α) activate β‐catenin to induce degeneration and apoptosis of NP cells (Hiyama, Yokoyama, Nukaga, Sakai, & Mochida, 2013; Wang et al., 2016). Moreover, the activation of β‐catenin by lithium chloride enhances the expression of Runx‐2 and MMPs and is involved in intervertebral disc senescence and calcification (Hiyama et al., 2010; Iwata et al., 2015).

β‐Catenin interacts with YAP1 to form a transcription complex which then regulates downstream gene expression. Activation of the Hippo pathway results in serine phosphorylation of YAP1 and its retention in the cytoplasm; then phosphorylated YAP1 binds to β‐catenin thereby preventing its translocation into the cell nucleus and leads to degradation (Imajo, Miyatake, Iimura, Miyamoto, & Nishida, 2012). On the other hand, tyrosine phosphorylation of YAP1 enhances its stability and prompts its interaction with β‐catenin and transportation into the nucleus (Rosenbluh et al., 2012). However, the role of YAP1/β‐catenin interaction in IDD remains unclear.

The aim of this study was to examine the regulatory role and mechanism of YAP1 in IL‐6‐induced IDD. We found that YAP1 overexpression significantly decreased Sox‐9, Col‐II, aggrecan expression, whereas increased MMP‐13 level in NP cells. Tyrosine but not serine phosphorylation of YAP1 was activated by IL‐6. Activated YAP1 interacted with β‐catenin and transported into nuclear to induce IDD development.

## 2 MATERIALS AND METHODS

### 2.1 Needle puncture induced IDD

All animal protocols were performed in accordance with the standard ethical guidelines and approved by Zhejiang University School of Medicine ethics committee.

Twelve‐week‐old Sprague Dawley rats were anesthetized using 2% pentobarbital sodium (0.2 ml/100 g), and the tail skin was disinfected with ethanol. The coccygeal 6/7 and 8/9 disks were punctured using a 21‐G needle and the 5/6 and 7/8 disks were used as sham. Then the rats were maintained under a 12/12 hr light/dark cycle and allowed free access to water and standard chow. Twelve weeks later, the rats were euthanized with an overdose of chloral hydrate, disks were isolated and preserved in liquid nitrogen.

### 2.2 Lumbar intervertebral disks ex vivo culture

Twelve‐week‐old Sprague Dawley rats were euthanized with an overdose of chloral hydrate, whole lumbar intervertebral disks including NP, annulus fibrosus (AF) and cartilaginous endplate (CEP) were isolated and cultured in Dulbecco's modified Eagle's medium (DMEM; Gibco, CA) containing 10% fetal bovine serum (FBS) (Gibco, CA) with 1% penicillin/streptomycin under 5% CO_2_ at 37°C. Then, the disks were randomized to three groups: (a) control group, (b) 100 ng/ml IL‐6 group, and (c) 100 ng/ml IL‐6 and 2 ug/ml verteporfin (VP) group. Seven days later, the disks were collected and analyzed by immunohistochemistry, the NP cells were isolated and detected by quantitative polymerase chain reaction (qPCR).

### 2.3 NP cell isolation and culture

NP cells were isolated from the lumbar disks of 12‐week‐old Sprague Dawley rats according to a previous report, with modifications (Hiyama et al., 2010). Brieﬂy, rats were euthanized with an overdose of chloral hydrate, and lumbar disks were excised. NP was isolated from the disks, washed with Hank's balanced salt solution, then minced and digested for 6 hr with 0.2 mg/ml collagenase II (Sigma‐Aldrich, San Francisco, CA), and the tissues were passed through a 100‐μm cell strainer (BD Biosciences, Franklin Lakes, NJ). The cells were collected and cultured in DMEM (Gibco, CA) containing 10% FBS (Gibco) with 1% penicillin/streptomycin under 5% CO_2_ at 37°C. The culture medium was changed every other day, and when confluent, the cells were harvested and subcultured in dishes. The cells within passage 6, which maintained NP cells morphology and phenotype, were used.

### 2.4 Transient transfection

NP cells were plated into six‐well plates 1 day before transfection. The next day, cells were transfected with YAP1 expression vector or small interfering RNA (siRNA) by using Lipofectamine 3000 (Invitrogen, Carlsbad, CA) as described by the manufacturer. After 48 hr, total proteins or RNAs were harvested from the transfected cells and subjected to western blot or qPCR analysis.

### 2.5 RNA isolation, RT‐PCR, and quantitative RT‐PCR

Total RNAs of NP cells were isolated by TRIZOL (Invitrogen) according to the manufacturer's protocols. The quantity of total RNAs was measured by Nanodrop 2000. Then, RNAs were reverse transcribed with PrimeScript RT Master Mix (Takara Bio, Otsu, Japan). qPCR was performed using SYBR Premix Ex TaqTM Kit (Takara, Dalian, China). The PCR cycling program was as follows: 95°C for 2 min, 40 cycles of 95°C for 10 s, 60°C for 20 s, and 72°C for 20 s. Melting curve analysis was carried out at the end of cycling program. The amplification signals from target genes were normalized by the glyceraldehyde‐3‐phosphate dehydrogenase (GAPDH) in the same reaction. All experiments were performed at least three times, and each sample was detected in triplicate.

### 2.6 Western Blotting

NP cells were placed on ice immediately after treatment and washed three times with ice‐cold phosphate‐buffered saline (PBS; Gibco). Cells were incubated for 20 min in radio‐immunoprecipitation assay (RIPA) buffer (Beyotime, China) supplemented with 100 mM phenylmethanesulfonyl fluoride (Beyotime, China), phosphatase inhibitor cocktail (Millipore), and Protease inhibitor cocktail (Millipore), followed by centrifugation at 12,000 rpm for 10 min to extract supernatant. Total proteins were separated on sodium dodecyl sulfate‐polyacrylamide gels and transferred by electroblotting to polyvinylidene fluoride (PVDF) membranes (Bio‐Rad, Hercules, CA). The membranes were blocked in 5%(w/v) non‐fat milk and then were detected with antibodies against the following: YAP1 (Cell Signaling Technology Cat# 4912 also 4912 S Lot# RRID:AB_2218911), S127‐YAP1 (Cell Signaling Technology Cat# 4911 S Lot# RRID:AB_2218913), Y357‐YAP1 (Abcam Cat# ab62751 Lot# RRID:AB_956486), Src (Abcam Cat# ab47405 Lot# RRID:AB_870739), Src phosphorylation (p‐Src; Abcam Cat# ab47411 Lot# RRID:AB_870740), β‐catenin (Abcam Cat# ab6301 Lot# RRID:AB_305406), and normalized to GAPDH (Abcam Cat# ab8245 Lot# RRID:AB_2107448).

### 2.7 Immunofluorescence staining

NP cells were plated on slides with appropriate density and incubated for 24 hr. The cells were treated with 100 ng/ml IL‐6, then fixed with 4% paraformaldehyde for 15 min, and permeabilized with 0.5% Triton X‐100. After blocking with 5% bovine serum albumin (BSA) for 30 min, slides were incubated with primary antibodies against YAP1 (1:100 dilution) and β‐catenin (1:100 dilution) for 2 hr. Then, the slides were washed three times with PBS and incubated with the Alexa Fluor 488/610‐conjugated secondary antibody and 4′,6‐diamidino‐2‐phenylindole for 1 hr. Samples were observed under confocal laser scanning microscopy.

### 2.8 Immunohistochemistry

Rat disc specimens were dehydrated with a graded series of ethanol, then embedded in paraffin, and cut into 4‐μm sections. To examine the expression of Col‐II and MMP‐13, sections were immunostained. Immunohistochemical analysis was performed using an SP Rabbit & Mouse HRP Kit (DAB; CW2069, CWBIO, China) according to the manufacturer's protocols. Rabbit anti‐Col‐II (Abcam Cat# ab34712 Lot# RRID:AB_731688) and MMP‐13 (Abcam Cat# ab39012 Lot# RRID:AB_776416) antibodies were used at a dilution of 1:200.

### 2.9 Statistical analysis

Expression levels of the genes were determined using the relative quantification method (2^−ΔΔCt^). All generated quantitative data were presented as the mean ± standard deviation, and diﬀerences were analyzed using one‐way analysis of variance. Statistical analyses were performed using SPSS 19.0. *p* < 0.05 was considered statistically significant for all tests.

## 3 RESULTS

### 3.1 Effects of IL‐6 on cultured NP cells

**Figure 1:**
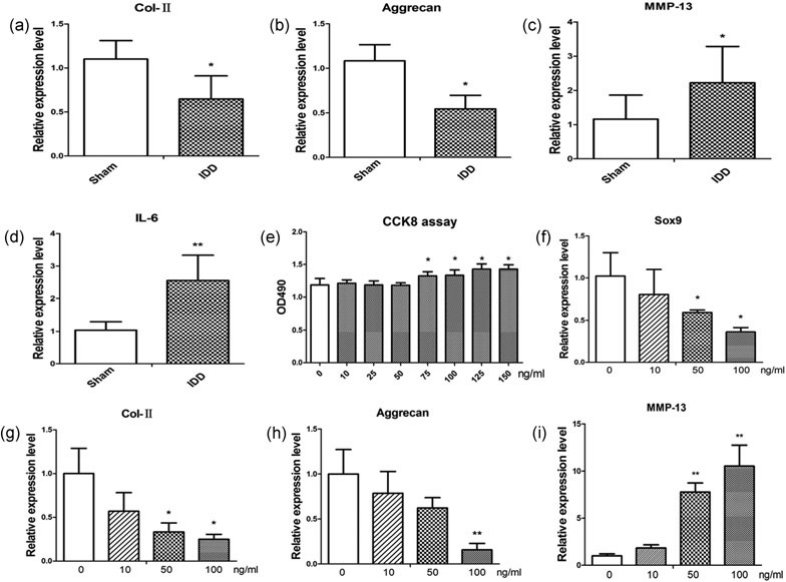
Effects of IL‐6 on NP cells. (a–d) mRNA expression level of Col‐II, aggrecan, MMP‐13, and IL‐6 in degenerative disks, *n* = 3. (e) NP cells were cultured with different concentration of IL‐6, after 48 hr CCK8 was performed to detect the cells viability, *n* = 5. (f–i) NP cells were cultured with different concentration of IL‐6; Sox‐9, Col‐II, aggrecan, and MMP‐13 were detected by qPCR after 48 hr, *n* = 3. Data represent means ± standard deviation of three independent experiments performed in triplicate. *p < .05, **p < .01 indicate significant differences between groups. CCK8: cell counting kit‐8; IL: interleukin; MMP‐13: matrix metalloproteinases 13; mRNA: messenger RNA

As shown in Figures 1a–d, Col‐II and aggrecan were decreased in induced degenerative rat disc, whereas MMP‐13 and IL‐6 were significantly increased. To examine the effects of IL‐6 on NP cells, cell counting kit‐8 (CCK8) was performed to evaluate cell viability. It showed that IL‐6 slightly increased NP cell viability (Figure 1e). Meanwhile, by IL‐6 treatment, expression of Sox‐9, Col‐II, and aggrecan was decreased, and MMP‐13 was increased in NP cells. These effects were evident at IL‐6 concentrations of 50 and 100 ng/ml (Figure 1f–i).

### 3.2 YAP1 was activated by IL‐6 in NP cells

**Figure 2:**
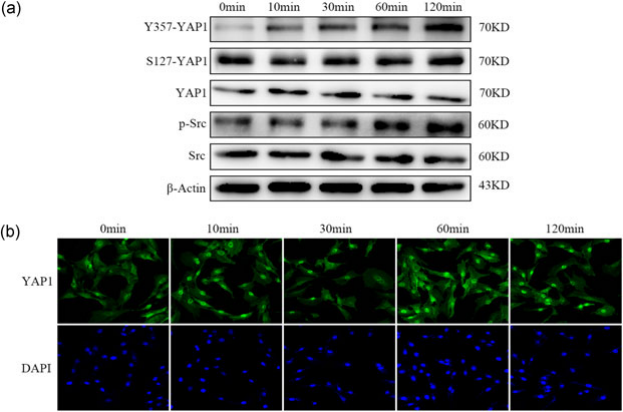
YAP1 was activated by IL‐6 in NP cells. (a) NP cells were cultured with 100 ng/ml IL‐6, western blot showed that Y357‐YAP1 and p‐Src were significantly upregulated (b) NP cells were cultured with 100 ng/ml IL‐6, immunofluorescence microscopy showed that YAP1 was transported into the nucleus. All experiments were performed at least three times, and each sample was detected in triplicate. IL: interleukin; p‐Src: Src phosphorylation; NP: nucleus pulposus; YAP1: yes‐associated protein 1 [Color figure can be viewed at wileyonlinelibrary.com]

As shown in Figure 2a, tyrosine phosphorylation of YAP1 (Y357‐YAP1) was significantly increased by IL‐6 treatment, but serine phosphorylation of YAP1 (S127‐YAP1) showed no significant difference. Immunofluorescence showed that IL‐6 promoted YAP1 nuclear translocation, indicating that YAP1 was activated by IL‐6 (Figure 2b). The previous study showed that YAP1 was activated upon tyrosine phosphorylation by tyrosine kinase Src (Taniguchi et al., 2015). Indeed, we found that IL‐6 elevated p‐Src in NP cells (Figure 2a). All these results indicated that IL‐6 enhanced YAP1 activation and stability.

### 3.3 Regulatory role of YAP1 on NP cells

**Figure 3:**
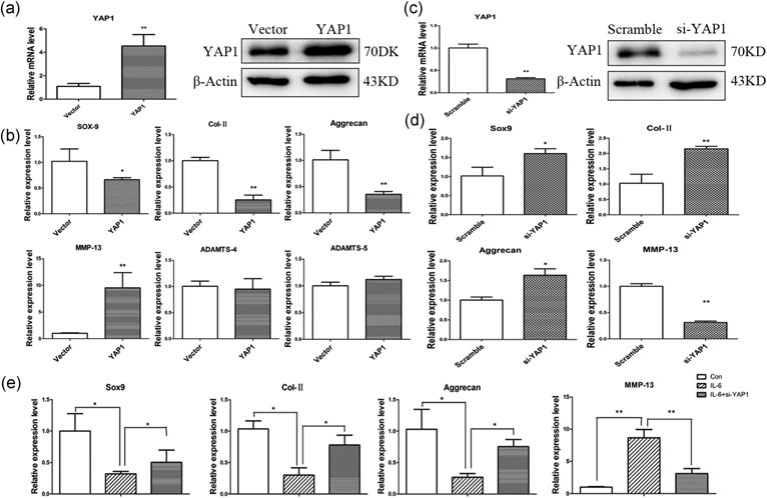
Regulatory role of YAP1 on NP cells. (a) NP cells were transfected with empty vector or YAP1 vector plasmid; overexpression efficiency was detected by qPCR and western blot after 48 hr. (b) NP cells were transfected with YAP1 vector; Sox‐9, Col‐II, aggrecan, MMP‐13, ADAMTS‐4, and ADAMTS‐5 were detected by qPCR after 48 hr. (c) NP cells were transfected with scramble sequence or YAP1 siRNA (si‐YAP1); qPCR and western blot showed YAP1 was knocked down in NP cells. (d) NP cells were transfected with si‐YAP1; and Sox‐9, Col‐II, Aggrecan, MMP‐13 were detected by qPCR after 48 hr. (e) NP cells were pretransfected with si‐YAP1 for 12 hr, then they were treated with IL‐6; and Sox‐9, Col‐II, Aggrecan, MMP‐13 were detected by qPCR after 48 hr. Data represent means ± standard deviation of three independent experiments performed in triplicate (*n* = 3). *p < .05, **p < .01 indicates significant differences between groups. ADAMTS: a disintegrin and metalloproteinase with thrombospondin motifs; MMP‐13: matrix metalloproteases 13; NP cells: nucleus pulposus cells; siRNA, small interfering RNA; YAP‐1: yes‐associated protein 1

We then investigated the effects of YAP1 on NP cells. First, we performed YAP1 overexpression in NP cells and the transfection efficiency was detected by qPCR and western blot (Figure 3a). As shown in Figure 3b, YAP1 overexpression significantly downregulated the expression of Sox‐9, Col‐II, and aggrecan, whereas increasing MMP‐13 levels. However, two other catabolic genes, ADAMTS‐4 and ADAMTS‐5, showed no significant difference. Then, YAP1 was knocked down by siRNA transfection (Figure 3c). We found that YAP1 knockdown significantly increased the expression of Sox‐9, Col‐II, and aggrecan, whereas decreased MMP‐13 expression (Figure 3d). To investigate the role of YAP1 in IL‐6‐induced NP cells degeneration, we pretransfected NP cells with siRNA for 12 hr, then treated cells with IL‐6. As shown in Figure 3e, YAP1 knockdown rescued IL‐6‐induced NP cells degeneration.

### 3.4 IL‐6 induced IDD through YAP1/β‐catenin signaling

**Figure 4:**
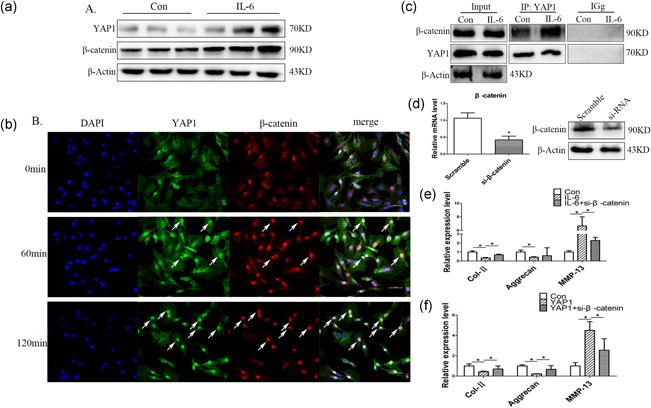
IL‐6 induced IDD through YAP1/β‐catenin signaling. (a) NP cells were cultured with or without 100 ng/ml IL‐6 for 48 hr, YAP1 and β‐catenin were detected by western blot. (b) NP cells were cultured with 100 ng/ml IL‐6, after 60 min or 120 min. The cells were fixed and stained with antibodies against YAP1 (green) and β‐catenin (red), and the nucleus was stained with DAPI (blue). (c) NP cells were cultured with or with 100 ng/ml IL‐6; Co‐IP was performed to detect the interaction between YAP1 and β‐catenin. (d) NP cells were transfected with scramble sequence or β‐catenin siRNA (si‐β‐catenin); qPCR and western blot showed that β‐catenin was knocked down in NP cells. (e) NP cells were pretransfected with si‐β‐catenin for 12 hr; then they were treated with IL‐6. Col‐II, Aggrecan, MMP‐13 were detected by qPCR after 48 hr. (f) NP cells were transfected with control plasmid, YAP1 plasmid or YAP1 plasmid and si‐β‐catenin for 48 hr; then Col‐II, Aggrecan, MMP‐13 were detected by qPCR. Data represent means ± standard deviation of three independent experiments performed in triplicate (*n* = 3). *p < .05, **p < .01 indicates significant differences between groups. DAPI: 4′,6‐diamidino‐2‐phenylindole; IDD: intervertebral disc degeneration; IL‐6: interleukin 6; MMP‐13: matrix metalloproteinases 13; NP cells: nucleus pulposus cells; siRNA: small interfering RNA; YAP1: yes‐associated protein 1 [Color figure can be viewed at wileyonlinelibrary.com]

Activated Wnt/β‐catenin signaling has been reported to promote IDD development (Wang et al., 2012; Wang et al., 2016). We found that after IL‐6 treatment for 48 hr, YAP1 and β‐catenin protein levels were significantly increased in NP cells (Figure 4a). Then, we investigated the effect of IL‐6 on YAP1 and β‐catenin protein cellular localization by Immunofluorescence. As shown in Figure 4b, IL‐6 promoted YAP1 (green) and β‐catenin (red) nuclear accumulation, and their colocalization (merged, yellow). Co‐IP further confirmed that IL‐6 enhanced the interaction between YAP1 and β‐catenin (Figure 4c). Moreover, when we knocked down the expression of β‐catenin in NP cells by siRNA (Figure 4d), and then treated the cells with IL‐6 or overexpressed with YAP1, we found that β‐catenin knockdown mostly rescued IL‐6 or YAP1‐induced NP cells degeneration (Figure 4e,f). These results suggested that IL‐6 induced degeneration of NP cells through YAP1/β‐catenin signaling, and β‐catenin was essential for the regulatory function of YAP1 in NP cells.

### 3.5 VP as a YAP1 inhibitor relieved IDD

**Figure 5:**
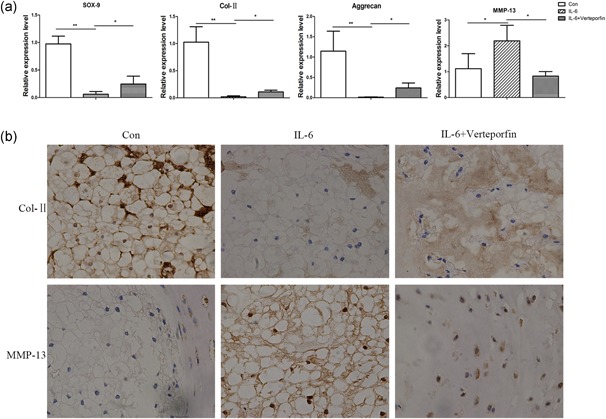
Verteporfin as a YAP1 inhibitor relieved IDD. Rat disks were cultured with PBS, IL‐6, or IL‐6 plus verteporfin for 7 days. (a) Sox‐9, Col‐II, aggrecan, MMP‐13 were detected by qPCR, *n* = 3. (b) Col‐II and MMP‐13 were detected by immunohistochemistry. *p < .05, **p < .01 indicates significant differences between groups. All experiments were performed at least three times, and each sample was detected in triplicate. IDD: intervertebral disc degeneration; IL‐6: interleukin 6; MMP‐13: matrix metalloproteinases 13; PBS: phosphate‐buffered saline; YAP1: yes‐associated protein 1 [Color figure can be viewed at wileyonlinelibrary.com]

Because VP has been recognized as an effective YAP1 inhibitor, we examined the effect of VP on IDD by ex vivo intervertebral disc culture. The results in Figure 5a showed that progression of IDD was alleviated by VP treatment by inhibiting the upregulation of MMP‐13 and increasing the expression of Sox‐9, Col‐II, and aggrecan. We further evaluated the effect of VP by immunohistochemistry; Col‐II and MMP‐13 were detected for they represent anabolic and catabolic genes, respectively. As shown in Figure 5b, VP partly rescued Col‐II expression and inhibited MMP‐13 expression. These results demonstrated that VP as a YAP1 inhibitor relieved IDD development.

## 4 DISCUSSION

As a multifactorial disease, the mechanism of IDD is complex and affected by various factors including mechanical stress, aging, inflammation, and infection (Adams, Freeman, Morrison, Nelson, & Dolan, 2000; Risbud & Shapiro, 2014; Wang, Cai, Shi, Wang, & Wu, 2016). IDD is characterized by degradation of the ECM and is associated with upregulation of inflammatory cytokines. IL‐6 is a well‐established proinﬂammatory cytokine that contributes to the pathogenesis of various degenerative diseases, and the role of IL‐6 as a key player in IDD has received attention recently. JAK/STAT3 is one of the well‐known pathways activated by IL‐6 and has been reported to induce IDD by increasing MMPs expression (Suzuki et al., 2016). However, the molecular mechanism of IL‐6‐induced IDD is not fully understood.

We hypothesized that YAP1 is involved in the regulation of IDD. Indeed, IL‐6 treatment activated YAP1 by elevating tyrosine phosphorylation but not by decreasing serine phosphorylation. The tyrosine kinase Src, as expected, was also activated. Furthermore, overexpression of YAP1 in NP cells induced ECM degradation by inhibiting Col‐II and aggrecan expression, and increasing MMP‐13 expression, although ADAMTS‐4 and ADAMTS ‐5 showed no significant changes. YAP1 knockdown showed opposite effects. Interestingly, the CCK8 assay showed that IL‐6 slightly elevated NP cell viability. Because YAP1 was a key regulator of cell contact inhibition and proliferation, the activation of YAP1 may account for the increased cell viability after treatment with IL‐6. Although slightly increased cell viability may have limited benefit to the intervertebral disc, we consider that the major role of YAP1 was to induce IDD development.

Wnt signaling typically involves a noncanonical pathway or a canonical pathway. Of these, the canonical WNT/β‐catenin pathway is well known. Wnt proteins form a dual receptor complex with Frizzled and low‐density lipoprotein receptor‐related protein 5 (LRP‐5) or LRP‐6 on cell surfaces to activate the signaling. This causes β‐catenin protein stabilization and translocation to the nucleus, where it binds to the lymphoid enhancer factor and T‐cell factor transcription factors to activate target gene expression (Lerner & Ohlsson, 2015). Wnt/β‐catenin signaling plays critical roles in bone and cartilage disease (Monroe, McGee‐Lawrence, Oursler, & Westendorf, 2012).

Here, we found that IL‐6 enhanced YAP1 and β‐catenin expression in NP cells. Immunofluorescence assay and Co‐IP showed that IL‐6 promoted their interaction and nuclear accumulation. We were interested in the role of β‐catenin in IL‐6/YAP1 signaling during IDD development. β‐catenin was knockdown and then NP cells were treated by IL‐6 or transfected with YAP1. We found that the downregulation of β‐catenin prevented ECM degradation. Therefore, we considered that β‐catenin was essential for IL‐6/YAP1 signaling‐induced IDD.

Due to the important role of YAP1 in IDD development, we supposed that it would be a potential target to treat IDD. YAP1 was inhibited by siRNA or VP, an FDA approved drug currently used for several angiogenic diseases in the clinic (Liu‐Chittenden et al., 2012), we found that inhibiting YAP1 partially reverse the degeneration caused by IL‐6 treatment. As we known, IL‐6 activated multiple pathways via receptor gp130, including JAK/STAT3, MAPK, and PI3K/Akt pathway (Garbers et al., 2012; Kishimoto, 2010). The activation of these pathways was also involved in apoptosis and inflammation of intervertebral disc (Li et al., 2017; Tian et al., 2013; Tu et al., 2017). Our data showed that inhibiting YAP1 incompletely rescued IDD which indicated that pathways independent of YAP1 may involve in IL‐6‐induced IDD.

One of the limitations in the current study was that we used rat but not human NP cells for in vitro research. Although rat NP cells were good alternatives to human for their extremely similar phenotype and function, a few differences still existed between them. NP cells, at least in part, were considered to be derived from notochordal cells (McCann & Seguin, 2016). Rat retains notochordal cells throughout much of its adult life but in human the number of notochordal cells decreases rapidly after birth. There were some inherent biochemical differences between human and rat; for example, rat aggrecan core protein lacks an extended keratan sulfate attachment domain and rat does not express MMP‐1 (Alini et al., 2008). Another limitation of our study was that we examined the effect of VP by ex vivo disc culture but not in vivo animal model. In summary, our results showed that YAP1 was activated by IL‐6 and accelerated ECM degradation in NP cells. β‐Catenin played a vital role in IL‐6/YAP1 signaling. The ex vivo study suggested that YAP1 may be a potential therapeutic target in IDD.
